# Comments on “Dynamic Adsorption of Sulfamethoxazole from Aqueous Solution by Lignite Activated Coke”

**DOI:** 10.3390/ma14040848

**Published:** 2021-02-10

**Authors:** Diego M. Juela

**Affiliations:** Department of Chemical Engineering, University of Cuenca, Cuenca 010107, Ecuador; diego.juela@ucuenca.edu.ec; Tel.: +593-984789005

**Keywords:** Bohart–Adams model, Yoon–Nelson model, Thomas model, Logistic model

## Abstract

This paper presents a brief discussion with regard to the fixed-bed modeling results of a recent paper by Li et al. published in this journal.

## 1. Introduction

The recent paper titled “Dynamic Adsorption of Sulfamethoxazole from Aqueous Solution by Lignite Activated Coke” published by Li et al. [1] in this journal describes dynamic column adsorption experiments to remove sulfamethoxazole (SMX) using lignite activated carbon. This paper is novel and has useful information in advancing the use of adsorption technology in the removal of emerging pollutants from wastewaters. However, there are two important points regarding the fixed bed modeling results which need to be discussed to avoid future confusion. 

## 2. Discussion

### 2.1. The Bohart–Adams Model 

In the Section 3.2 of their paper, the authors used the Bohart–Adams model labeled as “Adams-Bohart model” to fit the experimental data by linear regression and predict the parameters of the fixed-bed column. The linearized and breakthrough equation of this model used in their paper is given by Equations (1) and (2), respectively.

(1) $$\ln\left(\frac{C_{t}}{C_{0}}\right)=K_{A}C_{0}t-N_{0}h\frac{k_{A}}{v}$$

(2) $$Y=\exp\left(kt+b\right)$$

where, according to the authors, “*C*_0_ and *C_t_* (mg/L) are the concentration of the SMX solution in the inlet and outlet at time t (min), *k_A_* (L/(min·g)) is the Yoon–Nelson rate constant, *N*_0_ (g/L) is the sorption capacity of the adsorbent per unit volume of the bed, *h* (mm) is the column height, *v* (mm/min) is the flow rate”. However, in that statement, there are several typing errors. First, *h* is not the column height; it is the bed height. Second, *v* is not the flow rate; it is the superficial velocity of the SMX solution. Third, *k_A_* is not the Yoon–Nelson rate constant: it is the B-A rate coefficient [2]. There are also typing errors in Equations (1) and (2); specifically, the authors do not explain what *K_A_* is in Equation (1). It is clear that *K_A_* is the same parameter as *k_A_*. Although the authors do not explain what *Y, k,* and *b* are in Equation (2), by deduction it can be seen that:

(3) $$Y=\frac{C_{t}}{C_{0}}\;;\;\;\;\;\;\;\;\;k=K_{A}C_{0}t\;;\;\;\;\;\;\;\;b=\;N_{0}h\frac{k_{A}}{v}\;\;\;$$

Thus, the breakthrough equation should be written as Equation (4), and not as Equation (2). Note the sign error.

(4) $$\begin{array}{c} Y=\exp\left(kt-b\right)\\ \frac{C_{t}}{C_{0}}=\exp\left(K_{A}C_{0}t-N_{0}h\frac{k_{A}}{v}\right) \end{array}$$

Regarding the fixed bed modeling results, the authors obtained the worst fit to their experimental data with the “Adams-Bohart model”, with correlation coefficients R^2^ > 0.73. This low fit is due to the authors have used an oversimplified version of the Bohart–Adams model shown in Equations (1) and (2). The linearized and breakthrough equation of the original Bohart–Adams model is as shown in Equations (5) and (6) [3].

(5) $$\ln\left(\frac{C_{0}}{C_{t}}-1\right)=\frac{k_{A}N_{0}h}{v}-\;k_{A}C_{0}t$$

(6) $$\frac{C_{t}}{C_{0}}=\frac{1}{1+\exp\left(\frac{k_{A}N_{0}h}{v}-\;k_{A}C_{0}t\right)}$$

Equation (2) used by the authors in this study is an exponential function, and this increases proportionally with increasing time, thus this equation gives *C/C_0_* values higher than 1, which is illogical in adsorption processes. Equations (5) and (6) are clearly different from those used in this study. Equation (6) is a logistic function that predicts S-shaped breakthrough curves, and therefore this should be used to predict breakthrough curves in fixed-bed modeling studies. If the authors had used Equations (5) and (6) in their study, they would have obtained a better fit with the B-A model, and the conclusions could be different.

### 2.2. The Bohart–Adams, Yoon–Nelson and Thomas Model

The authors have used three equations—the Thomas model, the Yoon–Nelson model, and the Bohart–Adams model—to elucidate the adsorption mechanism and predict parameters of the fixed-bed column. However, the work of Chu [3] and other previous works [4,5,6] have demonstrated that these three models are mathematically equivalent to each other. Thus, the equations of these three models can be expressed by a single equation, called theLogistic model. The linearized and breakthrough equation of the Logistic model is shown in Equations (7) and (8), respectively.

(7) $$\ln\left(\frac{C_{0}}{C_{t}}-1\right)=a-\;bt$$

(8) $$\frac{C_{t}}{C_{0}}=\frac{1}{1+\exp\left(a-bt\right)}$$

**Figure 1:**
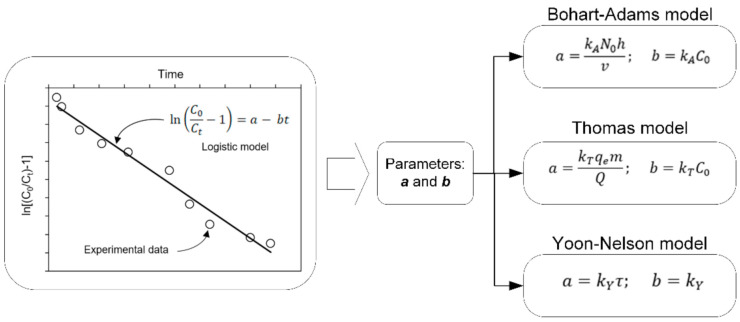
Correlations to estimate Bohart–Adams, Thomas, and Yoon–Nelson parameters.

Thus, the experimental data of the breakthrough curve should be fitted with the linear equation of the Logistic model, then the slope and intercept of the plot $\ln\left(\frac{C_{0}}{C_{t}}-1\right)$ vs *t* allow to obtain *b* and *a*, respectively. Finally, the parameters of the Thomas, Yoon–Nelson, and Bohart–Adams models can be computed using simple relations given in Figure 1. 

The authors state that the Thomas and Yoon–Nelson models are equivalent in terms of mathematical form, but despite that, they compare these two models and conclude that the Thomas model could best describe the column adsorption behavior. With the Logistic model, there is a single correlation coefficient value (R^2^), so it is not possible to compare the three models with their R^2^ value. Hence, it is not logical to compare the three models and say that one is better than the other since all three are mathematically equivalent. In that sense, the model comparison analysis presented by Li et al. [1] in their paper has flaws, and their conclusions are not meaningful. 

## Data Availability

Data is contained within the article.
